# Spotlight on the Nucleotide: Solid‐State NMR for the Investigation of ATP Hydrolysis in the ATPase SmsC

**DOI:** 10.1002/chem.71171

**Published:** 2026-05-28

**Authors:** Nina Wehr, Laure Decamps, Julius Schlüter, Koen Linssen, Georg Künze, Kushal Sengupta, Serena DeBeer, Thomas Wiegand

**Affiliations:** ^1^ Institute of Technical and Macromolecular Chemistry RWTH Aachen University Aachen Germany; ^2^ Max Planck Institute For Chemical Energy Conversion Mülheim an der Ruhr Germany; ^3^ Institute for Drug Discovery Leipzig University Leipzig Germany; ^4^ Interdisciplinary Center for Bioinformatics Leipzig University Leipzig Germany; ^5^ Center for Scalable Data Analytics and Artificial Intelligence Leipzig University Leipzig Germany

**Keywords:** ATP analogues, ATP hydrolysis, phosphorus‐31, real‐time NMR, solid‐state NMR

## Abstract

Solid‐state nuclear magnetic resonance (NMR) spectroscopy is an ideal tool to study ATP hydrolysis in ATP‐driven processes at atomic resolution. In this study, we present a nucleotide‐detected solid‐state NMR approach to probe nucleotide conformations and dynamics during ATP hydrolysis using the dimeric P‐loop ATPase SmsC as a model protein. ^31^P‐detected solid‐state NMR experiments under magic‐angle spinning (MAS) conditions provide insights into the different stages of ATP hydrolysis, which have been mimicked by ATP analogues that are often considered as “nonhydrolyzable”. Our data reveal that different degrees of conformational heterogeneity are observed for such ATP mimics. By computational modeling, we explore the role of a hydrogen bond between SmsC and the terminal phosphate group of the nucleotides in defining the nucleotide binding conformation. Homonuclear ^31^P‐^31^P dipolar coupling constant measurements for the bound ATP analogues have been performed to obtain information about their dynamic properties. These data show that the ATP and the transition‐state analogues maintain some molecular motion, while the post‐hydrolytic mimic AMPCP shows significantly less dynamics. The approach presented herein for investigating ATP hydrolysis can be easily transferred to other ATP‐fueled proteins, including difficult to express proteins or large protein complexes, therefore providing a way to functionally characterize these systems.

## Graphical abstract

Nucleotide‐detected solid‐state NMR has been applied to probe nucleotide conformations and dynamics during ATP hydrolysis using the dimeric P‐loop ATPase SmsC as a model protein. The different stages of ATP hydrolysis have been mimicked by ATP analogues for which different degrees of conformational heterogeneity have been observed.

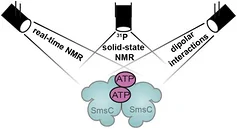

## 1 Introduction

Solid‐state nuclear magnetic resonance (NMR) spectroscopy is a powerful method for the investigation of ATP hydrolysis reactions, since structural as well as dynamic information of protein‐bound nucleotides are accessible at atomic resolution [1]. NMR permits the precise identification of the binding site and is even suitable to detect noncovalent interactions involved in binding, such as hydrogen bonding [2, 3, 4]. The possibility to gain highly resolved structural as well as dynamic information is a significant advantage over other techniques in the field of structural biology, such as X‐ray diffraction (XRD) or cryo‐electron microscopy (EM), for which it is currently difficult to obtain sub‐angstrom resolution needed for example to localize exact positions of hydrogen atoms involved in hydrogen bonding [5]. The power of solid‐state NMR in studying ATP hydrolysis has already been reported for several proteins including the bacterial helicase DnaB [2, 6, 7], the archaeal primase of the plasmid pRN1 [8], the ATP‐binding cassette (ABC) transporter BmrA [9], or the ATPase p97 [10, 11, 12], enabling to study the coupling of ATP hydrolysis with biological functioning.

**SCHEME 1:**
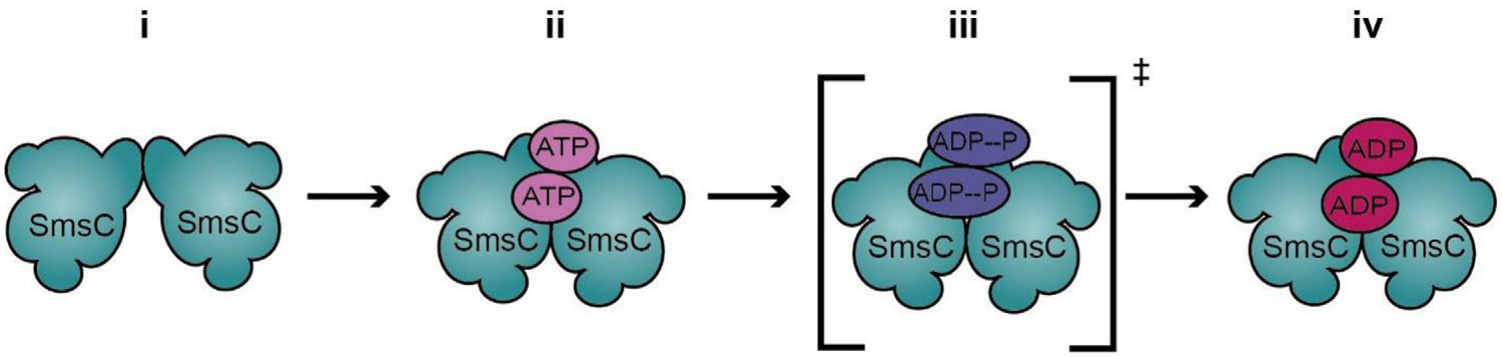
Schematic representation of the different stages occurring during enzyme‐catalyzed ATP hydrolysis here shown for the protein SmsC. The first stage represents the apo‐form of SmsC, where it is in an open conformation as observed by X‐ray crystallography in previous work [19]. Upon ATP binding (stage ii) the protein changes into a closed conformation [19]. During ATP hydrolysis the protein passes through a transition state (stage iii) and finally ends up in the post‐hydrolytic state where the reaction product ADP is bound to the protein.

The ATP hydrolysis process can be subdivided into four different stages (Scheme 1), which are (i) the apo state of the protein, where no nucleotide is bound, (ii) the pre‐hydrolytic ATP bound state, (iii) the transition state, where the P_β_‐O‐P_γ_ bond cleavage occurs and (iv) the post‐hydrolytic state where the hydrolysis product adenosine diphosphate (ADP) would be bound to the ATPase. Stages (ii) and (iii) are in most cases only short lived and for structural investigations using X‐ray crystallography, cryo‐EM or NMR, slowly or nonhydrolyzable nucleotide analogues need to be employed to trap stable snapshots of the hydrolysis reaction which mimic the physiologically‐relevant states as closely as possible [13]. The ATP in stage (ii) can be mimicked for instance by adenosine 5’ (β,γ‐imido)‐triphosphate (AMPPNP) or adenosine 5’ (β,γ‐methylene)‐triphosphate (AMPPCP) (Scheme S1), where the oxygen atom between the P_β_ and P_γ_ phosphate is replaced by an amine or methylene functionality, respectively [14, 15, 16]. The transition state of ATP hydrolysis (stage (iii)) can be mimicked by using, for example in vivo, which mimics an in‐line anionic transition state [17, 18]. A detailed spectroscopic investigation using solid‐state NMR and electron paramagnetic resonance (EPR) of this stage has been reported for DnaB [2]. Stage (iv) can again in most cases be assessed easily since in this stage ADP is bound to the protein, which is in many cases not hydrolyzed further [7].

In this study, we explore nucleotide‐detected solid‐state NMR spectroscopy aiming at shining light onto the conformation and dynamics of the nucleotide during the ATP hydrolysis cycle. The nucleotide‐detected NMR approach benefits from the fact that no specific protein isotope‐labelling and time‐consuming protein resonance assignment have to be performed to focus on enzyme‐catalyzed reactions, such as ATP hydrolysis. Instead, ^31^P is a rather sensitive NMR spin‐1/2 nucleus with a gyromagnetic ratio that is only a factor of around 2.5 smaller than the one of ^1^H and has a natural abundance of 100 %. Additionally, the large chemical‐shift dispersion makes it attractive for probing nucleotide conformations and their modulations upon protein binding [13, 20]. Changes in the typically large chemical‐shift anisotropy (CSA) provide insight into the dynamic properties of the nucleotide. It thus serves as a highly sensitive probe for nucleotide‐detected solid‐state NMR studies and could be applied in the case of low nucleotide concentrations, as for instance, occurring in large protein complexes or even in cells. A further advantage lies in the ease to follow ATP hydrolysis reactions in real time by using suitable ATP analogues. We illustrate this approach using the ATPase SmsC from the methanogenic archaea *Methanocaldococcus jannaschii*, examined here by solid‐state NMR for the first time, revealing unprecedented insights into nucleotide conformations during ATP hydrolysis and highlighting the broader utility of solid‐state NMR in studying the binding of small ligands to large biomolecules.

Our model protein SmsC belongs to the ABC‐type P‐loop ATPases [21]. It contains, in addition to the ABC‐signature motif, Walker A and Walker B motifs, which are known to be key in ATP hydrolysis [19, 22]. However, the exact mechanisms of ATP hydrolysis can vary in P‐loop ATPases and still need to be investigated for SmsC [23, 24]. One of the systems responsible for the biogenesis of Fe‐S clusters, which are crucial in various processes in life, for instance in the respiratory chain, is the Suf system [25]. This complex exists in different variations, always containing the proteins SufB and SufC, for example in *Escherichia coli* it is encoded as the operon *SufABCDSE*, while in *Bacillus subtilis* it is encoded as *SufCDSUB* [26]. The simplest version, consisting solely of the proteins SufB and SufC, can be found in methanogenic archaea [27]. It has therefore been classified as a minimal Suf‐system (Sms), and the Sms‐system was suggested to be an ancient homologue to the Suf‐system, which might even date back to the last universal common ancestor (LUCA) [27]. Recently, it has been found that in the Sms‐system the protein SmsC hosts the binding site for the Fe‐S cluster and additionally has an ATPase activity. Fe‐S cluster formation and ATP binding are mutually exclusive. This differs significantly from the Suf‐system, where SufB hosts the Fe‐S binding site [19]. The Suf or Sms‐system is of special interest, since it is not present in humans, but plays a central role for Fe‐S cluster biogenesis in various pathogens, such as the parasite *Blastocytis* [28], the malaria parasite *Plasmodium falciparum* [29], or *Staphylococcus aureus* [30] and *Mycobacterium tuberculosis* [31]. This makes it a promising target for the development of new pathogen‐specific drugs or antibiotics. A small targeting molecule has already been developed against *S. aureus*, which inhibits the Suf system [32].

## 2 Results and Discussion

### 2.1 Phosphorus‐31 Spectral Fingerprints Reveal Information on Conformational Heterogeneity

We initially aimed at proving the successful bacterial production strategy for SmsC by producing a uniformly ^13^C and ^15^N isotope‐labelled protein sample. Even though SmsC has a relatively low molecular weight of around 28 kDa (250 amino acid residues), it was possible to sediment the protein into the magic‐angle spinning (MAS) NMR rotor overnight by using a *g*‐force of 210.000 *g*, probably facilitated by the formation of dimers (or higher oligomers) at high protein concentrations observed previously for a variety of proteins [33, 34, 35]. Dimerization upon ATP binding is a well‐known behavior of the SufC protein in complex with SufB and SufD [22]. The obtained ^13^C‐detected 2D NMR spectra are well resolved (^13^C‐^13^C 2D dipolar assisted rotational resonance (DARR) [36] and ^15^N‐^13^C NCA spectra, Figure S1), pointing to a well‐folded protein conformation [37]. The same conclusion can be drawn from a proton‐detected NMR spectrum recorded at an MAS frequency of 100 kHz (for a discussion of such spectra see the Supporting Information). SmsC was found to possess comparably short bulk longitudinal relaxation times in the rotating frame (*T*_1ρ_), which complicates the acquisition of 3D experiments with a sufficient signal‐to‐noise ratio (SNR) due to rather inefficient CP polarization transfer steps (Figure S2). In the following, we present a nucleotide‐detected approach to study nucleotide‐bound states of SmsC using only natural abundance protein samples. SmsC‐nucleotide samples are prepared by simple sedimentation of protein samples overnight after incubating them with excesses of MgCl_2_ and nucleotide analogues.

**FIGURE 1:**
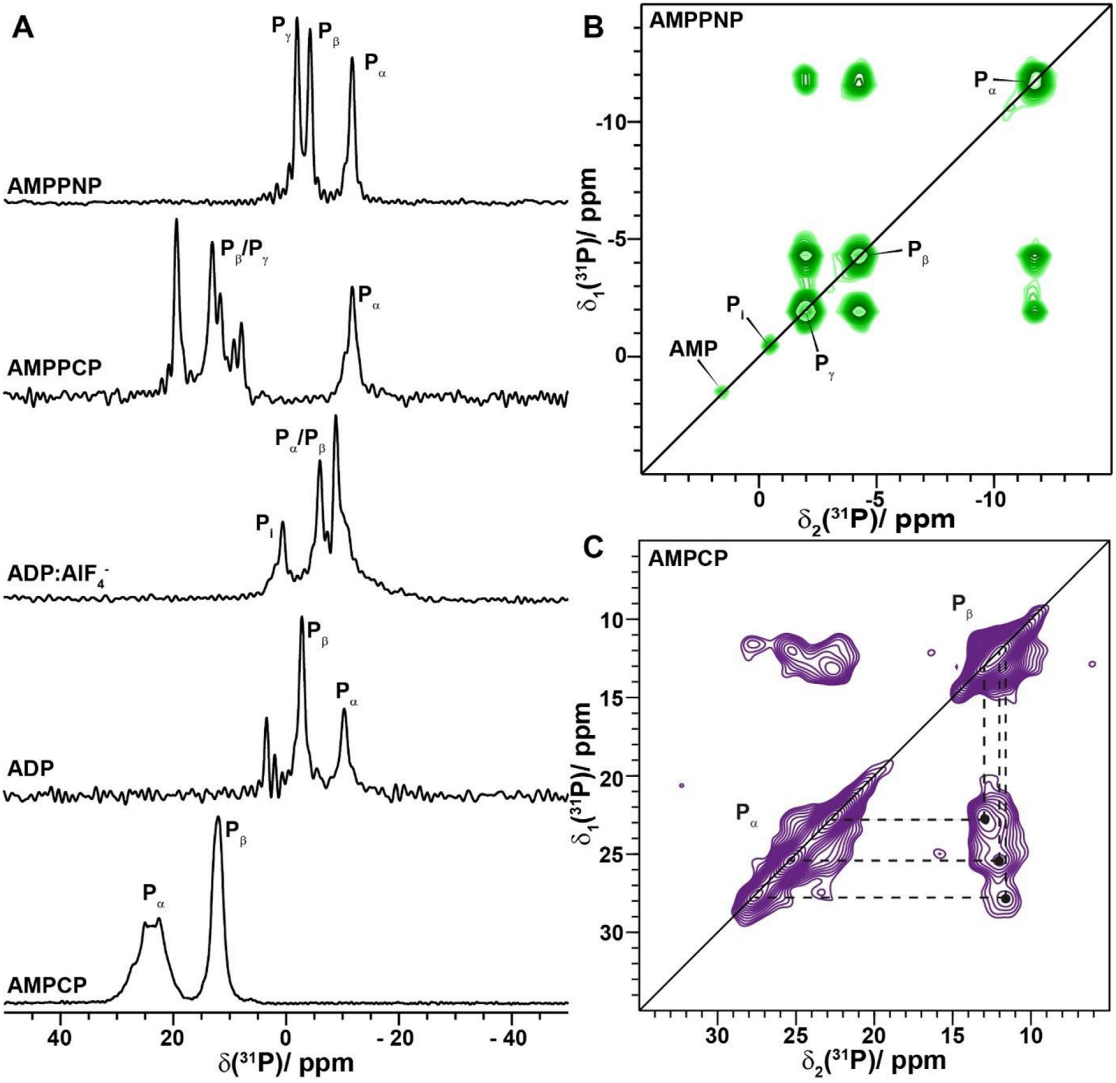
*AMPPNP and ADP bind in a single conformation to SmsC, whereas conformational heterogeneity is observed for AMPPCP and AMPCP*. (A) Stack plot of the ^1^H‐^31^P CP MAS NMR spectra from top to bottom of SmsC in complex with AMPPNP, AMPPCP, ADP:AlF_4_^−^, ADP and AMPCP. Except for the SmsC:ADP complex, all complexes were prepared with a ratio of 1:50 of SmsC:nucleotide. The SmsC:ADP complex was prepared using a ratio of 1:18 of SmsC:ADP (B) ^31^P‐^31^P DARR spectrum of the SmsC:AMPPNP complex recorded with a mixing time of 200 ms. (C) ^31^P‐^31^P DARR spectrum of the SmsC:AMPCP complex recorded with a mixing time of 200 ms. All spectra were recorded at 11.74 T using an MAS frequency of 17.0 kHz.

Trapping the initial state of ATP hydrolysis (the pre‐hydrolytic state) for SmsC, in which the triphosphate is bound to the protein, but no hydrolysis has occurred yet, has been achieved by using the ATP analogues AMPPNP [16] and AMPPCP [14] (Scheme S1 for the chemical structures). The first ^1^H‐^31^P CP‐MAS NMR spectrum of the SmsC:AMPPNP complex was taken four days after sample preparation (Figure 1A). Such a dipolar coupling‐based CP spectrum only reveals sufficiently immobilized (protein‐bound) nucleotides for which the CP polarization transfer is rather efficient [13, 38]. In this case, three rather sharp ^31^P resonances assigned to the three different phosphate groups of the triphosphate were observed (Figure 1A). Resonance assignment of these three resonances has been achieved based on a 2D ^31^P‐^31^P DARR spectrum (Figure 1B) in combination with previously reported ^31^P chemical‐shift values for AMPPNP in different protein systems with P_α_, P_β_, and P_γ_ resonating at −11.7 ppm, −4.2 ppm, and −1.9 ppm, respectively [6, 13, 39, 40]. These data point to a single well‐defined SmsC binding site for AMPPNP. Since SmsC exists in vivo in a complex with SmsB, it was probed whether the binding of AMPPNP to SmsC differs from the binding to the SmsCB complex. For this, a SmsCB:AMPPNP sample was prepared. The AMPPNP resonances in this sample overlay perfectly with the AMPPNP resonances in SmsC:AMPPNP (Figure S3) indicating that the binding mode of AMPPNP is highly similar in isolated SmsC compared to the protein complex SmsCB. Bound AMPPNP resonances are even narrower for SmsCB pointing to less conformational disorder.

In contrast, the SmsC:AMPPCP complex does not only reveal three distinct ^31^P resonances as expected, but at least two overlapping resonances assigned to the P_α_ phosphate and at least five different resonances that can be assigned to the P_β_ and P_γ_ phosphate groups were observed (Figure 1A). It was not possible to assign those resonances unambiguously to a specific phosphate, because the exact ^31^P chemical‐shift values of the P_β_ and P_γ_ resonances are significantly affected by the coordinating protein residues [13]. The different sets of AMPPCP resonances might occur due to different binding conformations of AMPPCP to SmsC.

Interestingly, in contrast to previous reports, such as for the bacterial DnaB helicase for which long‐term stable (order of several years) sedimented protein samples have been reported in case of various bound ATP mimics [35], both SmsC:nucleotide complexes are not stable over a time course of several weeks. In the SmsC:AMPPNP sample, 52 days after sample preparation and storing the MAS rotor in‐between at 4°C, additional ^31^P resonances appeared (Figure S4, note that no detailed time‐resolved stability tests have been performed). Those could be assigned to the hydrolysis products AMPPN at −10.5 ppm and −3.1 ppm, the free phosphate (P_i_) at 1.6 ppm and adenosine monophosphate (AMP) at 2.9 ppm. For the complex SmsCB a similar finding was made (Figure S3B). The formed AMPPN might either be formed by auto‐hydrolysis of the excess nucleotide in the supernatant or by enzymatic hydrolysis. Although a higher stability has been reported for AMPPCP compared to AMPPNP in several cases [13, 41], after 75 days, small spectral changes are observed as well, such as the appearance of a resonance assigned to a precipitated bisphosphonate species (PCP) [42] (Figure S4). To conclude, AMPPNP adopts a more defined binding conformation than AMPPCP, which clearly exhibits multiple binding conformations. This defined binding conformation makes AMPPNP the more suitable ATP analogue for further investigations of the pre‐hydrolytic state, despite its instability over time.

We next tried trapping the enzyme in a state closely resembling the ATP hydrolysis transition state by using ADP:AlF_4_^−^ as an ATP mimic [13, 17, 18]. The ^1^H‐^31^P CP‐MAS NMR spectrum of this sample features two ^31^P resonances at −6.0 ppm and −8.9 ppm that we assign to ADP:AlF_4_^−^ adopting a single binding conformation and a further resonance at 0.6 ppm, indicating the presence of P_i_ (Figure 1A). The assignment is further confirmed by a 2D ^31^P‐^31^P DARR spectrum (Figure S5). Similar ^31^P chemical‐shift values (P_α_ at −6.1 ppm and P_β_ at −7.4 ppm) have been reported for the complex of the bacterial DnaB helicase with ADP:AlF_4_^−^[7]. The resonances of ADP:AlF_4_^−^ also differ significantly from the resonances of the SmsC:ADP complex (*vide infra*, Table S1), which further supports the successful formation of the SmsC:ADP:AlF_4_^−^ complex (see also Figure S6 for the ^19^F MAS NMR spectrum). The P_i_ species observed in the spectrum could have been formed by enzymatic hydrolysis or during the preparation of ADP:AlF_4_^−^. In addition to the two sharp ^31^P resonances assigned to the SmsC:ADP:AlF_4_^−^ complex (Figure 1A), two broader resonances are detected. Those possibly arise from ADP, which coprecipitated with Al(OH)_3_ during the sample preparation [43].

For trapping the enzyme in the post‐hydrolytic state, we initially incubated SmsC with ADP. The formation of the SmsC:ADP complex was confirmed by the two ^31^P resonances in the ^1^H‐^31^P CP‐MAS NMR spectrum at −2.8 ppm and −10.4 ppm (Figure 1A). We verified the highly similar binding conformation of ADP in complexes with SmsC and SmsCB by comparing the ADP resonances of SmsC:ADP with those of SmsCB:ADP (Figure S7). The overlaying resonances indicate a similar binding conformation for both complexes. Additionally, a significant difference in the linewidth of the ^31^P resonances (smaller linewidth for SmsCB:ADP) was observed, pointing to less conformational flexibility in ADP binding of SmsCB compared to SmsC. A higher binding affinity has been reported before for the binding of ATP and analogues to SmsBC and the homologue Suf [19, 44]. Similar to the observations for AMPPNP, the SmsC:ADP complex is instable on the timescale of several days during which the sedimented protein sample was stored at 4°C (Figure S8). We next used adenosine (α,β‐methylene) diphosphate (AMPCP) [45] (Scheme S1) as a mimic for the post‐hydrolytic state, in which the P‐O‐P motif present in ADP is replaced by a P‐CH_2_‐P motif. SmsC:AMPCP reveals three distinct SmsC bound AMPCP conformations with ^31^P chemical‐shift values of 27.6 ppm, 25.4 ppm and 22.8 ppm (P_α_) and two resolved resonances at 12.8 ppm and 11.9 ppm (P_β_) (Figure 1A). The ^31^P resonance assignments were based on solution‐state NMR spectra of the free nucleotide [46]. These five different peaks show three different cross peaks in a 2D ^31^P‐^31^P DARR spectrum, supporting the presence of at least three different binding conformations of AMPCP (Figure 1C). The absence of the P‐O‐P bond also prevents the hydrolysis of the nucleotide by SmsC, as shown by ^31^P spectra recorded several weeks after rotor filling and storing the MAS NMR rotor with the sedimented protein sample at 4°C (Figure S8). The resolution of the spectra and the SNR even increase over time, the origin of which still requires further investigation. AMPCP thus acts as a long‐term stable ADP analogue when bound to SmsC, although a significant conformational heterogeneity is observed. We next sought to build models of the various SmsC:nucleotide complexes to obtain hints for the differences in the nucleotide‐binding behavior observed spectroscopically.

### 2.2 Structural Modelling of the Nucleotide‐binding Domain Points to the Importance of Hydrogen Bonding in Defining the Nucleotide Binding

**FIGURE 2:**
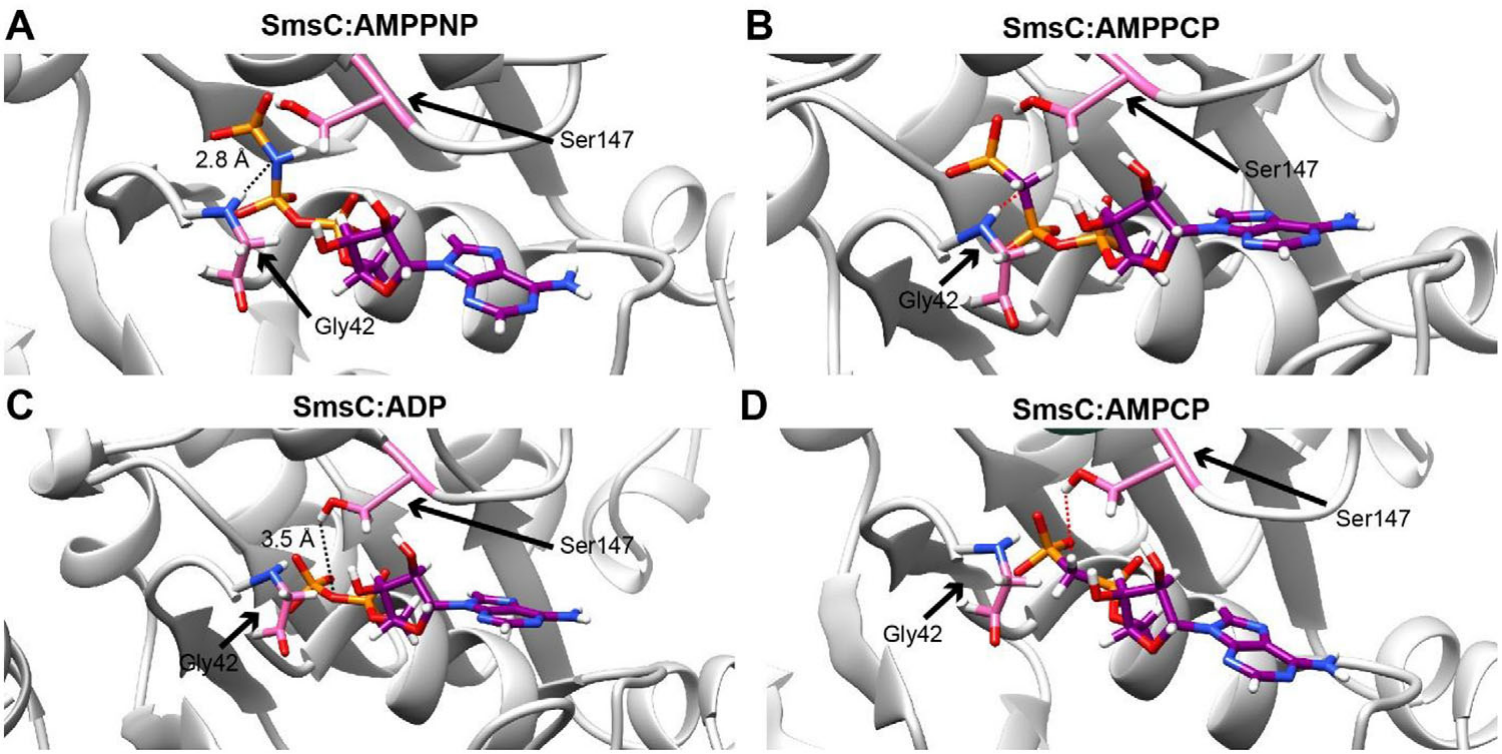
*The role of hydrogen bonding in defining ADP and AMPPNP binding in contrast to AMPCP and AMPPCP coordination*. Structural models of SmsC in complex with AMPPNP (A), AMPPCP (B), ADP (C) and AMPCP (D). The models were calculated based on the published single‐crystal structure of SmsC with AMPPN (PDB accession code 9H7X) [19]. The residue, which is identified to be involved in a hydrogen bond with the nucleotide based on such modelled structures is highlighted in purple and marked with an arrow. The postulated hydrogen bonds are indicated as black dashed lines and the hydrogen bond, which is not possible to form is indicated as red dashed lines. In (A) and (C) the distance of the possible hydrogen bond is indicated in Å.

Based on the recently published single‐crystal structure of SmsC with AMPPN (PDB access code 9H7X) [19], structures of SmsC with different bound nucleotides were modelled using local docking in the MOE software (see Experimental Section). The spectroscopic data was not included in such modelling. Starting from the cocrystallized AMPPN molecule, the other nucleotides were built in the binding pocket, followed by local induced fit docking, while the rest of the protein was kept fixed (Figure 2). The modelling work aims to identify possible reasons for the different binding conformations of AMPCP and AMPPCP as concluded from the ^1^H‐^31^P CP‐MAS NMR spectra. For this purpose, the model of SmsC:AMPPNP was compared to that of SmsC:AMPPCP and the model of SmsC:ADP to the one of SmsC:AMPCP. For the complexes SmsC:AMPPNP and SmsC:ADP, a single set of ^31^P resonances was observed in the CP spectra, which indicates a highly defined nucleotide binding conformation. For the model of SmsC:AMPPNP, a hydrogen bond between the backbone amide proton of Gly42 and the nitrogen bridging P_β_ and P_γ_ was assumed based on the observed spatial proximity (Figure 2A). This residue is located in the position K–3 in the Walker A motif, where it typically engages in a hydrogen bond with the oxygen atom bridging P_β_ and P_γ_ as well as an oxygen on the γ‐phosphate [24]. The formation of such a hydrogen bond is impossible for AMPPCP due to the inability of the CH_2_ group to act as a hydrogen bond acceptor (Figure 2B). This missing hydrogen bond might explain the conformational disorder of AMPPCP bound to SmsC observed experimentally, as its formation might be critical for this molecular recognition event.

For the modelled structure of SmsC:ADP, a possible hydrogen bond between Ser147 and the oxygen bridging P_α_ and P_β_ was identified (Figure 2C). Ser147 is a conserved residue in the LSGGQ ABC‐signature motif. Since the distance between the OH of Ser147 and the oxygen of ADP is 3.5 Å, slightly too long for a typical hydrogen bond, it could be bridged by a water molecule. The ABC‐signature motif is known to be involved in ATP binding in ABC‐transporters in a similar head‐to‐tail fashion as is observed for SmsC [47]. Again, the CH_2_‐unit in AMPCP is not able to function as a hydrogen bond acceptor (Figure 2D). The resulting missing hydrogen bond in the SmsC:AMPCP structure might again explain the observed conformational disorder of AMPCP, similarly to the one observed for AMPPCP.

*^31^P‐^31^P homonuclear dipolar coupling measurements reveal different molecular dynamics for ADP:AlF_4_^−^ and AMPCP bound to SmsC*

We then probed molecular dynamics of the bound nucleotides by quantifying the homonuclear ^31^P‐^31^P dipolar coupling strengths by solid‐state NMR experiments. To that effect, ^31^P‐^31^P Constant Time Dipolar Recoupling Effects Nuclear Alignment Reduction (CT‐DRENAR) experiments have been performed (to the best of our knowledge for the first time on protein nucleotide complexes), in which the ^31^P homonuclear dipolar coupling is recoupled under MAS conditions using the Back‐to‐Back (BaBa) double‐quantum (DQ) excitation scheme [48, 49, 50]. The homonuclear dipolar coupling constant between two nuclei relates in an isolated two‐spin system directly to the distance between the two nuclei via equation (1), where *b*_*jk*_ represents the dipolar coupling constant (in units of rad∙s^−1^) between the nuclei *j* and *k*, *μ_0_* the vacuum magnetic permeability, *γ* the gyromagnetic ratio and *r_jk_* the distance between the nuclei *j* and *k*.

(1) $$\;b_{jk}=-\frac{\mu_{0}}{4\pi }\frac{\hbar \gamma^{2}}{r_{jk}^{3}}$$

The dipolar coupling constant is determined from the experimental CT‐DRENAR curve by a fit to the first‐order approximation (equation (2)): [48]

(2) $$\;\frac{S_{0}-S^{\prime }}{S_{0}}=\frac{6}{5}\;{\left(b_{jk}NT_{r}\right)}^{2}\left(1+\cos\left(2\theta \right)\right)$$

with $S_{0}$ representing the reference signal, $S^{\prime }$ the dipolar‐dephased signal, θ the phase shift, and $NT_{r}$ the dipolar evolution period (Table S2). The expected distance between two phosphate groups in a di‐ or triphosphate is around 3 Å, as found in several P‐X‐P crystal structures as well as the crystal structure of ATP [14, 16, 51], which relates to a ^31^P‐^31^P dipolar coupling constant of ∼ –730 Hz. However, there are multiple factors that influence the measured effective dipolar coupling constant by CT‐DRENAR experiments, such as the ^31^P CSA that leads to lower (S_0_‐S’)/S_0_ values and thus an underestimation of the dipolar coupling. These factors can be empirically corrected following the procedures described in the Supporting Information (see also references [48, 52] for more details). Therefore, the determined dipolar coupling constants should not be translated into accurate distances in this case (as the CSA is quite substantial in some cases, *vide infra*), but their qualitative comparison for different ATP mimics reveals important insight into the dynamic properties of the protein‐bound nucleotides [53]. Since molecular motions significantly reduce the dipolar coupling interaction as well as the CSA, the combination of those two quantities can aid in investigating the dynamics of the bound nucleotides. We note that performing the CT‐DRENAR experiment at fast MAS frequencies can significantly reduce the influence of the CSA on the obtained dipolar coupling constants. Such experimental setups are currently being further investigated in our laboratory.

**FIGURE 3:**
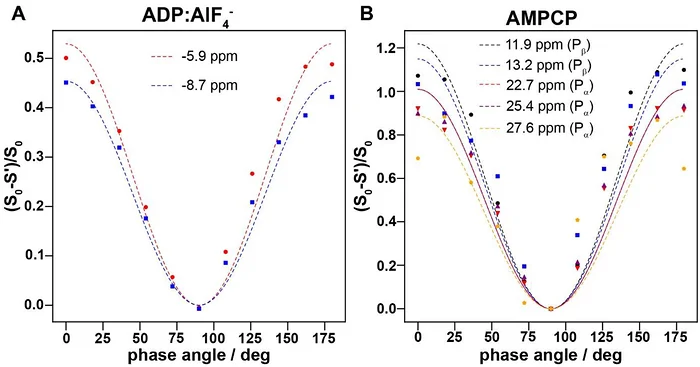
*^31^P‐^31^P CT‐DRENAR curves of different ATP analogues bound to SmsC can be used to measure homonuclear ^31^P‐^31^P dipolar coupling constants*. All CT‐DRENAR curves were recorded with a dipolar evolution time of 1.9 ms and are plotted as (S_0_‐S’)/S_0_ against the phase angle for the protein samples (A) SmsC complexed with ADP:AlF_4_^−^ and (B) SmsC complexed with AMPCP. The measured data points are given as symbols, while the fit is given as dashed lines. The CT‐DRENAR experiments were recorded at 11.74 T using an MAS frequency of 17.0 kHz.

When comparing the CT‐DRENAR curves of SmsC:AMPCP and SmsC:ADP:AlF_4_^−^, the highest respective (S_0–_S’)/S_0_ values at *θ* = 0° clearly indicate that the apparent dipolar coupling of AMPCP is significantly higher than that of ADP:AlF_4_^−^ (Figure 3A,B). Similarly, the ^31^P CSA of AMPCP determined from a slow‐spinning MAS spectrum is significantly higher than that of ADP:AlF_4_^−^ (Figures S9 and S10; Tables S3 and S4). The small CSA of SmsC:ADP:AlF_4_^−^ (reduced anisotropy, δ_σ_, of 0.05 kHz) is rather unexpected as a significantly higher ^31^P CSA has been determined, for instance for the same nucleotide bound to the bacterial helicase DnaB from *Helicobacter pylori* [2, 7] (Figure S11 and Table S5). Since a large CSA leads to an underestimation of the dipolar coupling constant, the corrected dipolar coupling constant of AMPCP is even larger than that of ADP:AlF_4_^−^ compared to the apparent difference in experimental curves (Table S2). The CT‐DRENAR curve, as well as the CSA, hint at some molecular motion of protein‐bound ADP:AlF_4_^−^ compared to AMPCP. The observed differences in molecular dynamics could be caused by a conformational change of SmsC during ATP hydrolysis. It is already established that both SmsC and SufC change their conformation during the ATP hydrolysis cycle from an open to a closed conformation [19, 22]. This behavior might also explain the observed changes in nucleotide dynamics. We attribute the higher molecular dynamics in the transition state (mimicked by ADP:AlF_4_^−^) to SmsC being in an open conformation and the reduced molecular dynamics in the post‐hydrolytic state (mimicked by AMPCP) to a closed conformation of SmsC. The pre‐hydrolytic SmsC:AMPPNP complex behaves similarly to the ADP:AlF_4_^−^ complex (e.g. a reduced ^31^P‐^31^P dipolar coupling constant is measured) and therefore, we hypothesize that AMPPNP also remains to some extent dynamic when bound to SmsC (Figure S12) and the protein possibly adopts an open conformation, which we will explore in future work.

### 2.3 The AMPPNP Hydrolysis Reaction in a Sedimented Sample Followed by Real Time NMR

**FIGURE 4:**
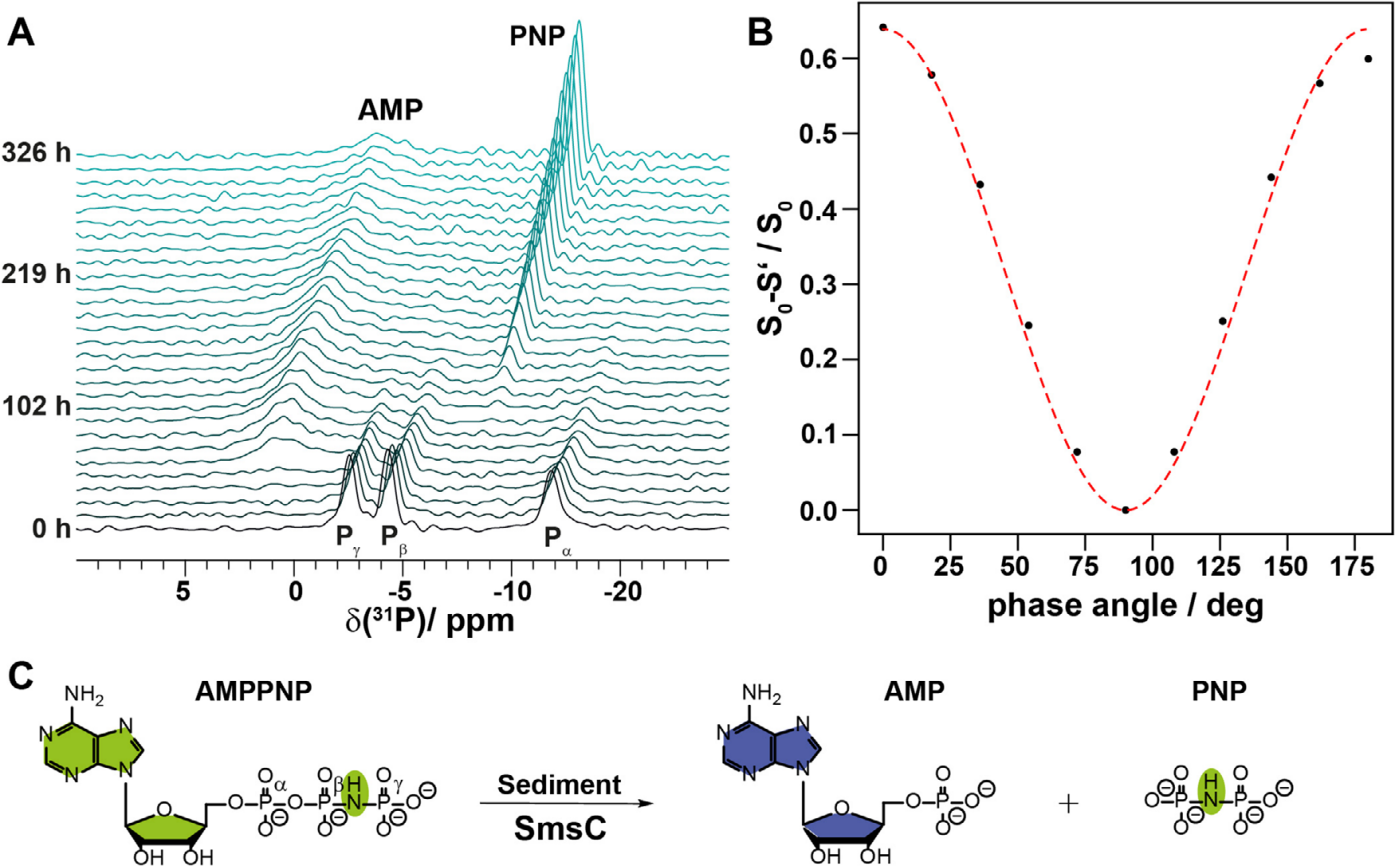
*Slowly hydrolyzing ATP analogues enable monitoring nucleotide hydrolysis by real‐time NMR spectroscopy*. (A) Time‐resolved ^1^H‐^31^P CP‐MAS NMR spectra of the AMPPNP hydrolysis process catalyzed by sedimented SmsC inside the MAS NMR rotor recorded at 11.74 T using a MAS frequency of 17.0 kHz and 286 K. The time resolution is around 10 h. (B) CT‐DRENAR curve recorded with a dipolar evolution time of 1.9 ms for the ^31^P resonance assigned to PNP observed in the kinetic experiment measured 9 months after storage at 4°C. The dashed red line represents the fit according to Ren and Eckert [49] indicating a dipolar coupling constant of −548 ± 4 Hz (the error only contains the statistical uncertainty). The resulting dipolar coupling constant has not been corrected for inaccuracies arising from finite pulse length or the CSA. The experiment was recorded at 11.74 T using an MAS frequency of 17 kHz. (C) Schematic representation of the hydrolysis reaction of AMPPNP (green) to AMP (dark blue) catalyzed by SmsC at 303 K occurring inside the solid‐state NMR rotor.

Finally, we wondered whether the slowly hydrolysable ATP analogues offer the unique possibility to follow the hydrolysis of the nucleotides in real time by NMR. Real‐time solid‐state NMR experiments of ATPases have been performed previously, for instance, for the ABC‐transporters LmrA [54] and MsbA [55], the ATPase p97 [10], a diacylglycerol kinase [56] as well as the archaeal pRN1 primase [42]. In that vein, we decided to explore the pre‐hydrolytic ATP analogue AMPPNP. In contrast to the previously shown spectra (Figure 1), real‐time NMR spectra on SmsC were taken at a sample temperature of 30°C to accelerate nucleotide hydrolysis and enable its spectroscopic observation on the time scale of several days of NMR measurement time (Figure 4). Since SmsC originates from the thermophilic archaea *M. jannaschii*, the protein does not undergo denaturation at ambient temperatures. Time‐resolved ^1^H‐^31^P CP‐MAS NMR spectra on a sedimented protein sample have been collected (Figure 4A).

Surprisingly the observed reaction products are AMP at 1.6 ppm and imidodiphosphate (PNP) at –7.5 ppm (Figure 4A). The AMP resonance is significantly broadened, pointing to an unspecific adsorption on the protein surface. The sharp PNP signal hints at a precipitation in complex with Mg^2+^, which is present in the reaction mixture. To further confirm that this single resonance belongs to the two symmetric phosphate groups in PNP, the homonuclear dipolar coupling constant of this ^31^P resonance was determined by recording a CT‐DRENAR [48] curve (Figure 4B). If this resonance is indeed PNP, it should exhibit a ^31^P dipolar coupling constant of around –720 Hz, which would correspond to the previously reported distance of ∼3 Å [14, 16]. The measured dipolar coupling of −548 Hz was slightly smaller than expected and would point to a P‐P distance of 3.3 Å. The reason for this underestimated dipolar coupling is most likely the ^31^P CSA, which was not further considered in the data analysis. This finding agrees with recent observations on the dinucleotide formation reaction catalyzed by the archaeal pRN1 primase, during which bisphosphonate (PCP) is formed whose precipitation in the MAS NMR rotor in complex with Mg^2+^ has been reported [42]. The protein thus catalyzes the hydrolysis from AMPPNP to AMP and PNP under the experimental conditions used in the MAS NMR rotor (Figure 4C), while in solution, no sign of PNP formation was found, neither in the NMR spectra nor in the form of a visible precipitate in the NMR tube. Instead, hydrolysis of AMPPNP to AMPPN and P_i_ is observed, which is catalyzed by SmsC (Figure S13, for the contribution of AMPPNP auto‐hydrolysis see Figure S14). The reason for differences in the hydrolysis behavior between sediment and solution might be associated with different protein concentrations (300 vs. 24 mg/mL, respectively), the effect of MAS and subtle temperature differences. The hydrolysis of the P‐O‐P bond of AMPPNP is only very rarely observed for ATPases, but has been reported before, for example for snake venom phosphodiesterase [16]. Instead the hydrolysis of the P‐N‐P bond in AMPPNP is rather common and has been observed for a multitude of proteins before for example, by the motor domain of the protein ncd [39], the sarcoplasmic reticulum Ca^2+^‐ATPase [57], the helicase DnaB [6] or by beef heart mitochondrial ATPase [58].

## 3 Conclusions

**FIGURE 5:**
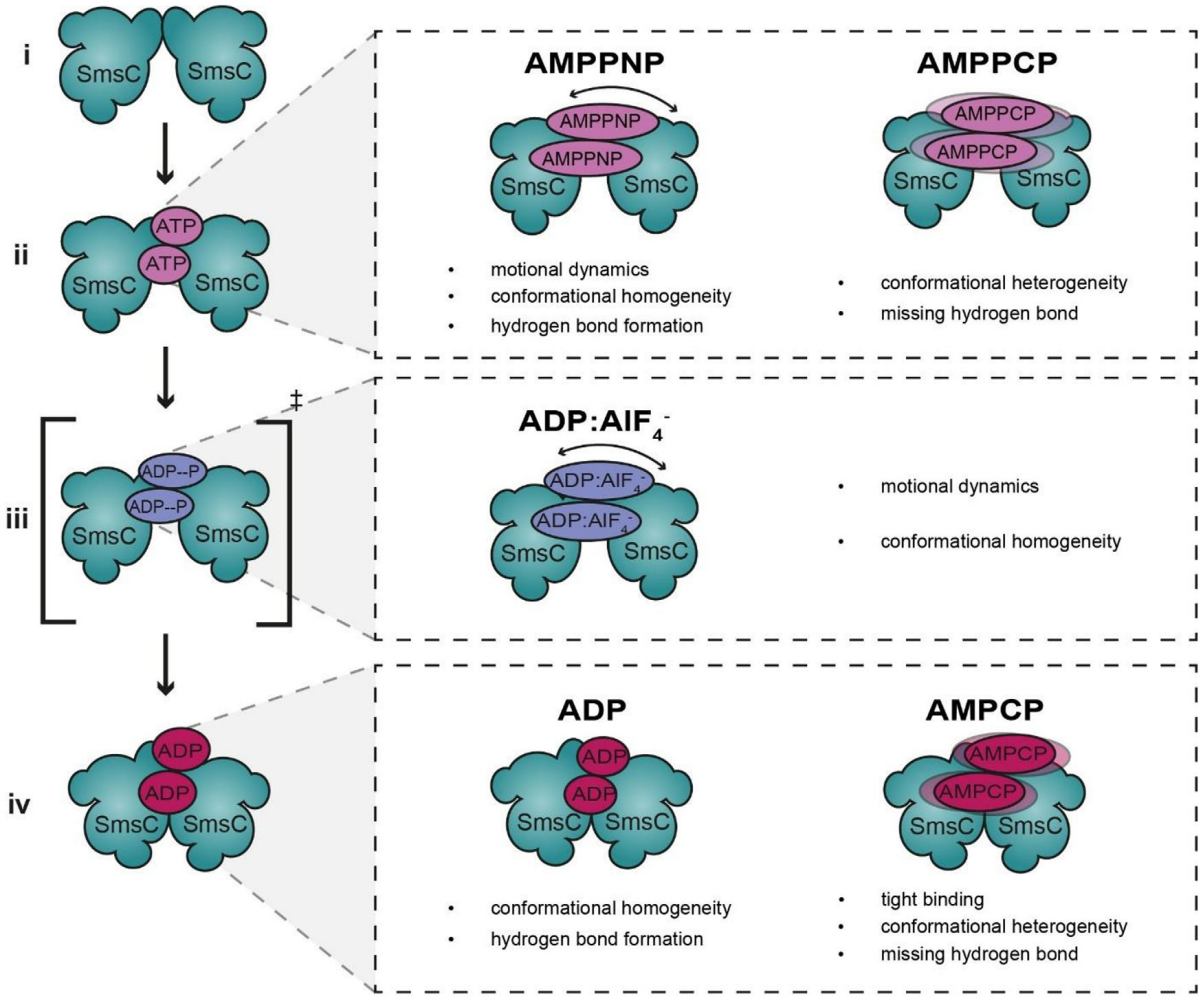
*Schematic summary of the different binding behaviors observed for SmsC*. Shown on the left are the different hydrolysis stages that were investigated in this study. The hypothesized change from an open to a closed conformation after the transition state (stage iii) is indicated. For each stage, the utilized nucleotides during this study are indicated, as well as their respective binding behavior.

We have applied herein a nucleotide‐detected solid‐state NMR approach to follow ATP hydrolysis catalyzed by the ATPase SmsC by exploring ATP analogues that mimic different stages in the ATP hydrolysis reaction as closely as possible (Figure 5). Conformational heterogeneity in nucleotide binding has been observed for the pre‐hydrolytic ATP mimic AMPPCP and the post‐hydrolytic ATP analogue AMPCP, whereas a single well‐defined binding conformation has been found for AMPPNP, ADP:AlF_4_^−^ and ADP. The latter SmsC:nucleotide complexes, however, are prone to hydrolysis. With the help of structural modelling, the importance of a hydrogen bond to the atom bridging the terminal phosphate groups has been identified in achieving a well‐defined nucleotide binding conformation. If the hydrogen bond formation is not possible, as in the case of AMPCP and AMPPCP, multiple binding conformations of the nucleotide have been detected. To further relate binding heterogeneity to nucleotide mobility, we have employed ^31^P‐^31^P homonuclear dipolar coupling measurements using for the first time the CT‐DRENAR pulse scheme to probe nucleotide molecular dynamics. Differences in mobility of the bound nucleotides during ATP hydrolysis might be associated with conformational changes of the protein during the hydrolysis cycle, for example the change from an open to a closed conformation. Kinetic information on the hydrolysis of AMPPNP has been obtained by real‐time NMR on sedimented SmsC protein samples revealing the hydrolysis of AMPPNP between P_α_ and P_β_ in contrast to hydrolysis in solution where the cleavage occurs between P_β_ and P_γ_. The herein presented nucleotide‐detected approach clearly benefits from not requiring expensive ^13^C/^15^N isotope labelling strategies, as well as time‐consuming sequential resonance assignment processes to monitor enzyme‐catalyzed reactions, which is especially useful for proteins that require expression in mammalian cells, where isotope labelling is still challenging. Also, large proteins or complexes there‐of benefit from this method, since for those cases, sequential resonance assignment becomes increasingly difficult or even entirely impossible. The smart incorporation of additional NMR active nuclei such as ^2^D or ^19^F in the ATP mimics will pave the way for further promising applications in nucleotide‐detected solid‐state NMR spectroscopy.

## Conflicts of Interest

The authors declare no conflicts of interest.

## Supporting information




**Supporting File**: The authors have cited additional references within the Supporting Information [59, 60, 61, 62, 63, 64, 65, 66, 67, 68, 69, 70, 71, 72, 73, 74, 75, 76, 77, 78, 79].

## Data Availability

The data that support the findings of this study are openly available in Radar4Chem at https://www.radar‐service.eu/radar/en/dataset/9epqdh2vq50unfz0?token = ZCczCxHlFfBXPbCQEJme.
