# Supplementary material for: Spotlight on the Nucleotide: Solid‐State NMR for the Investigation of ATP Hydrolysis in the ATPase SmsC
Source: Chemistry. 2026 May 28;32(34):e71171. doi: 10.1002/chem.71171 (PMC13569932; doi:10.1002/chem.71171)
Supplement: Supplementary file 1 [file CHEM-32-e71171-s001.docx]

**Supplementary Information for**

**Spotlight on the nucleotide: Solid-state NMR for the investigation of ATP hydrolysis in the ATPase SmsC**

Nina Wehr^a,b^, Laure Decamps^b,*^, Julius Schlüter^a^, Koen Linssen^a,b^, Georg Künze^c,d,e^, Kushal Sengupta^b^, Serena DeBeer, and Thomas Wiegand^a,b,*^

^a^ Institute of Technical and Macromolecular Chemistry, RWTH Aachen University, Worringerweg 2, 52074 Aachen, Germany

^b^ Max Planck Institute for Chemical Energy Conversion, Stiftstr. 34-36, 45470 Mülheim/Ruhr, Germany

^c^ Institute for Drug Discovery, Leipzig University, 04103 Leipzig, Germany

^d^ Interdisciplinary Center for Bioinformatics, Leipzig University, 04107 Leipzig, Germany

^e^ Center for Scalable Data Analytics and Artificial Intelligence, Leipzig University, 04105 Leipzig, Germany

*: Corresponding authors. L.D.: laure.decamps@protonmail.com; T.W.: thomas.wiegand@cec.mpg.de

**Materials and Methods**

*Production and Purification of SmsC and SmsCB*

SmsC as well as SmsCB were produced in *Escherichia coli BL21* transformed with pRSF-Duet-1 containing the *smsC* gene or both the *smsC* and *smsB* genes overnight in LB medium at 37 °C after induction with IPTG at OD 0.6-0.8. SmsC was His-tagged and SmsB was Strep-tagged. The cell pellet was resuspended in a buffer containing 50 mM Tris, 150 mM NaCl at pH 7.5. Afterwards the cells were lysed via sonication. The cell lysate was centrifuged for 1 h at 135.700 *g* and the supernatant was incubated under stirring in a 48 °C water bath for 20 min. The solution was centrifuged at 8.000 *g* and the supernatant was filtered with 45 µm filters. The sample was loaded on a 5 mL HisTrap Cytiva column, after preequilibration of the column with a buffer containing 50 mM Tris, 150 mM NaCl at pH 7.5. For elution of the protein a gradient with 50 mM Tris, 150 mM NaCl, 500 mM imidazole over 20 column volumes was used. The fractions containing the target protein were concentrated and dialyzed in a buffer containing 25 mM Tris, 50 mM NaCl at pH 7.5 and loaded onto a size exclusion column (26x60) and eluted using the same buffer. The fraction containing the target protein was concentrated and the purity was verified by running an SDS-PAGE. The protein was quantified using the Lowry assay.^[1]^

*Expression of uniformly ^13^C/^15^N labelled SmsC*

For labeling of the protein with ^13^C or ^15^N, *E. coli BL21* cells transformed with pRSF-Duet-1 containing the *smsC* gene were grown in M9 minimal medium^[2]^ containing ^13^C sucrose as a sole carbon source or ^15^N ammonium as a sole nitrogen source, respectively, and the same procedures as the production and purification of the protein from cells grown in LB medium were followed.

*Expression and preparation of the uniformly ^13^C/^15^N labelled DnaB sample*

The protein DnaB was expressed according to the previously reported protocol.^[3]^ The preparation of the DnaB:ADP:AlF_4_^–^ complex was performed analogous to the procedure published by Wiegand *et al*..^[4]^

*Preparation of the nucleotide complexes*

For the preparation of the nucleotide complexes with AMPPNP, AMPPCP and AMPCP (obtained from Jena Bioscience), a nucleotide stock solution and a MgCl_2_ stock solution in 25 mM Tris buffer with 50 mM NaCl and adjusting the pH-value to 7.5 was prepared. The stock solutions were afterwards added to the SmsC solution to achieve a ratio of SmsC: nucleotide: MgCl_2_ of 1:50:50. For the ADP complex was prepared in a similar fashion, except the achieved ratio was 1:18:18. For the preparation of the ADP:AlF_4_^–^complex first a NH_4_AlF_4_ solution was prepared by mixing a solution AlCl_3_ with NH_4_F in a molar ratio of 1:5. Afterwards a MgCl_2_ solution, the NH_4_AlF_4_ solution and the ADP solution were added stepwise to the SmsC solution to achieve a ratio of SmsC:MgCl_2_: NH_4_AlF_4_:ADP of 1:50:50:50. During this procedure the pH was kept between 6 and 7.5 to prevent precipitation of SmsC or Al(OH)_6_. After an incubation time of 1 h at 4 °C the samples were sedimented into the MAS rotor over night with a *g*-force of 210.000 *g* by using a Beckman Coulter Optima XPN 80 ultracentrifuge.

*Solid-state NMR experiments*

The solid-state NMR experiments on the uniformly ^13^C/^15^N labelled SmsC sample were performed at a static magnetic-field strength of 16.4 T equipped with a Bruker Avance III console. The ^13^C-detected experiments were performed using a MAS frequency of 17.0 kHz with a Bruker Biospin 3.2 mm triple resonance E-free probe, while the ^1^H-detected 2D ^1^H-^15^N hNH experiment was performed using a MAS frequency of 100.0 kHz with a Bruker Biospin 0.7 mm triple resonance probe. The ^31^P-detected experiments of the protein-nucleotide complexes were performed at a static magnetic field of 11.74 T with a Bruker Avance III HD console using a MAS frequency of 17.0 kHz or 3.0 kHz with a Bruker Biospin 3.2 mm triple resonance probe. For full details of the solid-state NMR experiments see Table S6.

*Real-time NMR experiments*

For the solid-state NMR real-time experiment, the protein-AMPPNP complex was prepared as described above. The exact amount of protein inside the MAS NMR rotor was determined to be 14.2 mg by measuring the protein concentration before the sample preparation using the UV absorbance at 280 nm and by measuring the protein concentration in the supernatant after centrifugation by performing a BCA assay. The exact amount of AMPPNP in the MAS NMR rotor was determined to be 12.7 µmol by measuring the amount of nucleotide in the supernatant after sedimentation using ^31^P solution-state NMR. During preparation, the sample was constantly handled at 4 °C where only very slow turnover of AMPPNP is assumed. The sample temperature in the NMR experiment was calibrated for 30 °C by using the H_2_O resonance (variable temperature unit (VTU) temperature at 286 K). The time point zero was set to the first solid-state NMR experiment measured at 30 °C. The experiments were performed at a static magnetic-field strength of 11.74 T with a Bruker Avance III HD console using an MAS frequency of 17.0 kHz with a Bruker Biospin 3.2 mm triple resonance probe.
For the solution-state NMR real-time experiment, the SmsC:AMPPNP complex was prepared similar as described above by using 14.2 mg SmsC and 12.7 µmol AMPPNP and MgCl_2_ in 600 µL 25 mM Tris buffer with 50 mM NaCl with 10 % D_2_O. The experiments were performed at a static magnetic-field strength of 14.1 T with a Bruker Avance Neo spectrometer.

*Structural modelling*

The X-ray crystal structure of the dimeric form of SmsC2 from *M. jannaschii* (PDB ID:9H7X) bound to AMP phosphoramidate (AMPPN) was used as the starting model. The structure was energy-minimized and protonated using the **QuickPrep** tool in MOE (Chemical Computing Group ULC, Montreal, QC, Canada, 2025). The different adenine nucleotides (ADP, ATP, ATPγS, AMPCP, AMPPCP, AMPPN, AMPPNP) were constructed based on the AMPPN structure using the MOE Builder module and subsequently docked into the receptor using the **Triangle Matcher** placement algorithm. The top 100 docking poses were refined with the **Induced Fit** protocol, and final poses were scored using the **GBVI/WSA dG** scoring function implemented in MOE.^[5]^ Docked conformations were finally ranked according to their S-scores.

**Sedimented SmsC reveals highly resolved solid-state NMR spectra**

In addition to the solid-state NMR experiments at 17 kHz, we have also performed experiments in an 0.7 mm rotor, allowing the acquisition of ^1^H-detected spectra at an MAS frequency of 100.0 kHz. As discussed in detail previously, the coherent homogeneous broadening typically dominating the experimentally observed proton linewidth for protein samples reduces significantly upon increasing the MAS frequency.^[6–8]^ The ^1^H-detected 2D ^1^H-^15^N cross-polarization (CP)-based hNH spectrum shows decently resolved peaks with amide proton linewidths in the order of 160 Hz (full-width-at-half maximum, FWHM), which is of similar magnitude as for other fully protonated protein samples at 100 kHz MAS (Figure S1C).^[7,9–14]^ The observed linewidth for a selected set of resonances in proton, carbon and nitrogen dimensions (160 ± 14 Hz, 120 ± 10 Hz and 96 ± 9 Hz, respectively) are all within an expected range for a well-folded protein and thus indicate the successful expression and purification of an SmsC sample highly suitable for solid-state NMR studies (Figure 1D).

The simple sample preparation by sedimentation^[3,15]^ is a further advantage of solid-state NMR over XRD or cryo-EM, where single crystals or proteins in a diversity of orientations on the EM grid have to be produced.^[16–18]^ This holds in particular also for the formation of protein-ligand complexes as explored in detail in this work

**Discussion of the influences on dipolar couplings determined by CT-DRENAR experiments**

There are certain factors, which influence the measured dipolar coupling (b_app_). Those are e.g., dipolar truncation, the chemical-shift anisotropy (CSA) and local dynamics of the nucleotides. Dipolar truncation means it is not possible to measure weak dipolar coupling in the presence of a strong dipolar coupling.^[19]^ In a multi-spins-system it is only possible to measure an effective dipolar coupling, which is a combination of all present dipolar couplings.^[20,21]^ In a triphosphate the dipolar coupling between P_α_ and P_γ_ can therefore not be determined, since it is much smaller than the dipolar coupling of P_α_ to P_β_ or P_γ_ to P_β_. Since a triphosphate is not a two-spin system, it is also not possible to determine the distance from P_β_ to P_α_ or P_γ_ based on the dipolar coupling of P_β_, because it experiences the dipolar coupling to both. A large CSA also leads to a less accurate determination of the dipolar coupling. In general, a larger CSA leads to an underestimation of the dipolar coupling. Exchange processes generate molecular motion, which makes the dipolar coupling less effective. This would also lead to an underestimation of the dipolar coupling. Additionally, there are experimental limitations, such as finite pulse length, which lead to an error in the determination of the dipolar coupling. It is possible to correct for those errors with empirical correction factors leading to the corrected dipolar coupling constants *b*_corr_.^[22]^

**Description of the fit used for the analysis of the CT-DRENAR experiments**

For the analysis of the CT-DRENAR data the determined integrals were plotted as (S_0_-S’)/S_0_ over the phase angle, which is varied during the CT-DRENAR experiment.^[23]^ Afterwards the data was fitted with the equation (1) as described by Ren and Eckert^[22]^,

$$\frac{S_{0}-S'}{S_{0}}= \frac{6}{5}\left( b\cdot t_{r} \right)^{2}(1+cos(2\theta))$$

(1)

where *b* is the dipolar coupling constant, *t_r_* the evolution time and *θ* the phase angle. The obtained value from the fit is afterwards corrected for the approximations of using finite pulse length (*f_p_*) and the CSA, according to the equations (2) and (3), where *t_p_* is the pulse length of a π/2 pulse, a is a factor depending on the true dipolar coupling constant, *δ*_aniso_ is the reduced anisotropy in Hz and *ν*_MAS_ is the MAS frequency.

$$f_{p}=1-2.53\left( \frac{t_{p}}{0.5 t_{r}} \right)$$

(2)

$$f_{c}=1-a\left( \frac{\delta_{aniso}}{\nu_{MAS}} \right)^{2}$$

(3)

The correction factors are then applied to the dipolar coupling constant obtained from the fit of equation (1), by using equation (4) to obtain the corrected dipolar coupling constant *b_corr_*.

$$b_{corr}=\frac{b}{\sqrt{f_{p}\cdot f_{c}}}$$

(4)

**Scheme S1**: *Chemical structures of ATP-mimics used in this study.* The employed nucleotides can be used to model the pre-hydrolytic state (AMPPNP and AMPPCP), the transition state (ADP:AlF_4_^–^) and the post-hydrolytic state (ADP and AMPCP).

**Figure S1**: *Sedimented SmsC gives highly resolved solid-state NMR spectra.* (**A**) Aliphatic region of a 20 ms 2D ^13^C-^13^C DARR spectrum of ^13^C/^15^N isotope labelled SmsC recorded at 16.4 T using a MAS frequency of 17.0 kHz. (**B**) 2D ^15^N-^13^C NCA spectrum of SmsC recorded at 16.4 T using a MAS frequency of 17.0 kHz. (**C**) 2D ^1^H-^15^N hNH spectrum of fully protonated SmsC recorded at 16.4 T using a MAS frequency of 100.0 kHz. (**D**) FWHM linewidths (total linewidths) of ^1^H, ^13^C and ^15^N obtained from the 2D ^1^H-^15^N hNH, 2D ^13^C-^13^C DARR and 2D ^15^N-^13^C NCA spectra respectively. For each boxplot the linewidth of ten different isolated peaks was used. The blue line in the boxplot indicates the median of the data. The peaks were randomly chosen across the entire spectrum and are encircled in red in (**A**), (**B**) and (**C**).

**Figure S2**: *The bulk T_1ρ_ relaxation times of ^1^H and ^13^C for the proteins SmsC and DnaB differ.* Bulk ^13^C *T_1ρ_* relaxation times of SmsC and DnaB were determined to 2.4 ± 0.01 ms and 3.9 ± 0.04 ms, respectively. The bulk ^1^H *T_1ρ_* relaxation times of SmsC and DnaB were determined to 2.0 ± 0.01 ms and 3.0 ± 0.02 ms, respectively. All experiments were performed at a magnetic-field strength of 16.4 T with a spin-lock nutation frequency of 45 kHz and 17.0 kHz MAS frequency.

**Figure S3**: *AMPPNP bound to SmsC and to SmsCB possesses the same ^31^P chemical-shift values and hydrolyzes similarly.* (**A**) ^1^H-^31^P CP MAS-NMR spectra of SmsC:AMPPNP (green) and SmsCB:AMPPNP (turquoise) recorded directly after sample preparation for solid-state NMR. (**B**) ^1^H-^31^P CP MAS-NMR spectra of SmsCB:AMPPNP recorded 4 (light turquoise) and 32 days (dark turquoise) after sample preparation. All spectra were recorded at a static magnetic-field strength of 11.7 T and an MAS frequency of 17.0 kHz.

**Figure S4**: *AMPPNP is hydrolyzed by SmsC within few weeks, while AMPPCP shows only minor sign of hydrolysis.* (**A**) ^1^H-^31^P CP-MAS NMR spectra of SmsC with AMPPNP in a ratio of 1:50 recorded four days (dark green) and 52 days (light green) after sample preparation, the cyan labels mark the resonances belonging to AMPPN and the purple labels mark the resonances of AMPPNP. (**B**) ^1^H-^31^P CP-MAS NMR spectra of SmsC with AMPPCP in a ratio of 1:50 recorded two days after sample preparation (dark cyan) and 75 days after sample preparation (light cyan). All spectra were recorded at 11.74 T using an MAS frequency of 17.0 kHz.

**Figure S5:** *The ^31^P-^31^P DARR spectrum confirms the assignment of ADP:AlF_4_^–^.* Shown is a 2D ^31^P-^31^P DARR spectrum of SmsC with ADP:AlF_4_*^–^* with a mixing time of 20 ms recorded at 11.74 T using a MAS frequency of 17.0 kHz.

**Figure S6**: *^19^F EASY spectrum reveals the formation of ADP:AlF_4_^–^.* ^19^F MAS-NMR spectrum of SmsC:ADP:AlF_4_*^–^* recorded with EASY background suppression. The peaks marked with ° have been assigned to precipitated AlF_x_(OH)_6-x_ species. The spectrum was recorded at a magnetic-field strength of 11.7 T and an MAS frequency of 17.0 kHz.

**Figure S7**: *ADP bound to SmsC and to SmsCB possesses the same ^31^P chemical-shift values.* ^1^H-^31^P CP MAS-NMR spectrum of SmsC:ADP (cyan) and SmsCB:ADP (purple). Both spectra were recorded at a magnetic-field strength of 11.7 T and an MAS frequency of 17.0 kHz.

**Figure S8**: *A stable post-hydrolytic state of SmsC was formed by using the ADP-mimic AMPCP, since SmsC hydrolyses ADP further to AMP.* (**A**) ^1^H-^31^P CP-MAS spectra of SmsC with ADP in a ratio of 1:18 recorded four days (dark blue) and 32 days (light blue) after sample preparation (**B**) ^1^H-^31^P CP-MAS spectra of SmsC with AMPCP in a ratio of 1:50 recorded four days after sample preparation (dark purple) and 23 days after sample preparation (light purple) recorded at 11.74 T using a MAS frequency of 17.0 kHz.

 **Figure S9**: *Determination of ^31^P CSA parameters of SmsC:AMPCP.* The ^1^H-^31^P CP MAS-NMR spectrum was recorded at 11.7 T with an MAS frequency of 3.0 kHz. The line shape simulation is given in red. ° indicates center bands and * indicates sidebands. The fits of the individual resonances are stacked on top and labelled on the left. The δ_iso_ value of the individual resonances is given on the right. The results of the fit are given in Table S3.

 **Figure S10**: *Determination of ^31^P CSA parameters of SmsC:ADP:AlF_4_^–^.* The ^1^H-^31^P CP-MAS NMR spectrum was recorded at 11.7 T with an MAS frequency of 3.0 kHz. The line shape simulation is given in red. The fits of the individual resonances are stacked on top and labelled on the left. The results of the fit are given in Table S2. Note that the isotropic resonances of ADP:AlF_4_^–^ differ from the ones observed at 17.0 kHz MAS, which might be due to differences in the sample temperature.

**Figure S11**: *Determination of ^31^P CSA parameters of DnaB:ADP:AlF_4_^–^:DNA.* The ^1^H-^31^P CP MAS-NMR spectrum was recorded at 11.7 T with an MAS frequency of 3.0 kHz. The line shape simulation is given in red. The fits of the individual resonances are stacked on top and labelled on the left according to ^[4]^ The resonance at -9.3 ppm is assigned to an unidentified component, which is possibly a degradation product and was included for better fitting results. The results of the fit are given in Table S4

**Figure S12:** *^31^P CT-DRENAR curves of AMPPNP bound to SmsC can be used to measure homonuclear ^31^P-^31^P dipolar coupling constants.* The CT-DRENAR curve was recorded with an evolution time of 1.9 ms and plotted as (S_0_-S’)/S_0_ against the phase angle. The measured data points are given as symbols, while the fit to the first-order approximation of the dephasing is given as dashed lines. The results of the fit are given in Table S2. The CT-DRENAR experiments were recorded at 11.74 T using a MAS frequency of 17.0 kHz.

**Figure S13:** *In solution a different hydrolysis reaction of AMPPNP is observed than in the sediment.* (**A**) Time-resolved ^31^P solution-state NMR spectra of SmsC with AMPPNP at 30 °C, recorded at 14.1 T. The time resolution is around 10 h. The resonances labelled in green belong to the educt AMPPNP, the resonances labelled in orange belong to the product AMPPN. (**B**) Schematic representation of the hydrolysis reaction of AMPPNP catalyzed by SmsC at 303 K occurring inside the solution-state NMR tube.

**Figure S14:** *Auto-hydrolysis of AMPPNP has only a small influence on the AMPPNP hydrolysis kinetics catalyzed by SmsC in solution.* (**A**) First and last ^31^P solution-state NMR spectra after 1 h and 90 h of the control experiment to account for AMPPNP auto-hydrolysis. (**B**) Normalized intensity of the P_i_ signal (as P_i_ / (P_i_ + P_α_)) over the reaction time of the AMPPNP hydrolysis with addition of SmsC (red) and without SmsC (blue). For the control experiment without SmsC a concentration of 15.8 mM AMPPNP and MgCl_2_ in 25 mM Tris buffer with 50 mM NaCl and pH 7.5 was used. The experiment was performed at 303 K at a static magnetic field of 14.1 T.

**Table S1:** Overview of ^31^P isotropic chemical-shift values determined from 1D ^1^H-^31^P CP MAS-NMR spectra recorded at 17 kHz and the corresponding assignment of all resonances. If the assignment is ambiguous, the shifts were allocated to all possible species.

| Sample | AMP / ppm | P_i_ / ppm | Other / ppm | P_α_ / ppm | P_β_ / ppm | P_γ_ / ppm |
| --- | --- | --- | --- | --- | --- | --- |
| AMPPNP day 4 | - | - | - | -11.7 | -4.2 | -1.9 |
| AMPPNP day 52 | 2.9 | 1.6 | - | -11.8 / -10.5 | -4.3 / -3.1 | -2.0 |
| SmsCB:AMPPNP |  |  |  | -11.6 | -4.2 | -2.0 |
| AMPPCP day 2 | - | - | - | -11.7 / -10.5 | 19.4 / 13.1 / 11.7 / 9.2 / 7.9 | |
| AMPPCP day 75 | 1.6 | - | 21.3 (PCP) | -11.7 / -10.5 | 19.4 / 13.1 / 11.7 / 9.2 / 7.9 | |
| ADP:AlF_4_*^–^* day 2 | - | 0.6 | - | -6.0 / -8.9 | | - |
| ADP day 4 | 3.4 | 2 | - | -10.4 | -2.8 | - |
| ADP day 32 | - | 2 | - | -10.4 | -2.8 | - |
| SmsCB:ADP |  |  |  | -10.4 | -2.8 |  |
| AMPCP day 4 | - | - | - | 27.0 / 25.1 / 22.5 | 12.0 | - |
| AMPCP day 23 | - | - | - | 27.6 / 25.4 / 22.8 | 12.8 / 11.9 | - |
| AMPPNP kinetic start | - | - | - | -11.8 | -4.3 | -2.5 |
| AMPPNP kinetic end | 1.7 | - | -7.5 (PNP) | - | - | - |

**Table S2**: ^31^P-^31^P dipolar coupling constants obtained from the experimental CT-DRENAR curves using the fit described in equation (1). *b*_app_ is the fitted dipolar coupling, *b*_corr_ is the fitted dipolar coupling after correction for approximation of finite pulse length and the CSA according to equation (2) and (3) and Ren et al.^[22]^ and *b*_diff_ is the difference between *b*_corr_ and the expected dipolar coupling of 729 Hz, *r* is the distance calculated based on *b*_corr_ according to equation (1). In the sample the nucleotide is always understood in complex with SmsC.

| Sample | Peak / ppm | *b*_app_ / Hz | *b*_corr_ / Hz | *b*_diff_ / Hz | *r* / Å |
| --- | --- | --- | --- | --- | --- |
| SmsC:AMPPNP | –11.7 (P^α^) | –241 ± 6 | –250 ± 6 | 479 | 4.29 |
| SmsC:AMPPNP | –4.2 (P^β^) | –333 ± 4 | –346 ± 4 | 383 | 3.85 |
| SmsC:AMPPNP | –1.9 (P^γ^) | –259 ± 4 | –269 ± 4 | 460 | 4.19 |
| SmsC:ADP:AlF_4_*^–^* | –8.9 | –231 ± 3 | –240 ± 3 | 450 | 4.13 |
| SmsC:ADP:AlF_4_*^–^* | –6.0 | –250 ± 5 | –260 ± 5 | 428 | 4.03 |
| SmsC:AMPCP | 11.9 (P^β^) | –378 ± 10 | –601 ± 16 | 128 | 3.20 |
| SmsC:AMPCP | 13.2 (P^β^) | –368 ± 12 | –563 ± 18 | 166 | 3.27 |
| SmsC:AMPCP | 22.7 (P^α^) | –344 ± 8 | –553 ± 13 | 176 | 3.29 |
| SmsC:AMPCP | 25.4 (P^α^) | –345 ± 9 | –532 ± 14 | 197 | 3.33 |
| SmsC:AMPCP | 27.6 (P^α^) | –323 ± 17 | –481 ± 25 | 248 | 3.45 |

**Table S3:** Results of the fit of the ^31^P CP MAS-NMR spectrum of SmsC:ADP:AlF_4_*^–^* recorded at a static magnetic field strength of 11.7 T with an MAS frequency of 3.0 kHz. *δ*_iso_ is the isotropic chemical shift, *δ_σ_* the reduced anisotropy, and *η* the asymmetry according to the Haeberlen-Spiess-Mehring convention.^[24–26]^

| *δ*_iso_ / ppm | *δ_σ_* / kHz | *η* |
| --- | --- | --- |
| –10.0 | 0.05 | 0.15 |
| –11.2 | 0.45 | 0.14 |
| 0.4 | –4.7 | 0.96 |
| –7.8 | 13.4 | 0.87 |

**Table S4:** Results of the fit of the ^31^P CP-MAS spectrum of SmsC:AMPCP recorded with an MAS frequency of 3.0 kHz. *δ*_iso_ is the isotropic chemical shift, *δ_σ_* the reduced anisotropy and η the asymmetry according to the Haeberlen-Spiess-Mehring convention.^[24–26]^

| *δ*_iso_ / ppm | *δ_σ_* / kHz | *η* |
| --- | --- | --- |
| 11.9 | 13.1 | 0.57 |
| 13.3 | 12.7 | 0.61 |
| 22.8 | –13.2 | 0.95 |
| 25.4 | 12.8 | 0.94 |
| 27.8 | 12.4 | 0.93 |

**Table S5:** Results of the fit of the ^31^P CP-MAS spectrum recorded of DnaB:ADP:AlF_4_*^–^* recorded with an MAS frequency of 3.0 kHz. *δ*_iso_ is the isotropic chemical shift, *δ_σ_* the reduced anisotropy and *η* the asymmetry according to the Haeberlen-Spiess-Mehring convention.^[24–26]^

| *δ*_iso_ / ppm | *δ_σ_* / kHz | *η* |
| --- | --- | --- |
| 0.2 | –24.3 | 0.76 |
| –1.3 | –25.0 | 0.76 |
| –6.1 | –29.0 | 0.49 |
| –7.3 | 24.7 | 0.39 |
| –9.3 | –7.3 | 0.74 |

**Table S6:** Overview of the experimental parameters of the solid-state NMR experiments. For more information about the adiabatic CP steps and the used tangential shapes see reference ^[27]^.

| **Sample** | **SmsC UL ^13^C/^15^N** | **SmsC UL ^13^C/^15^N** | **SmsC UL ^15^N** | **SmsC UL ^13^C/^15^N** |
| --- | --- | --- | --- | --- |
| **Experiment** | **^13^C-^13^C DARR** | **^15^N-^13^C NCA** | **^1^H-^15^N hNH** | **^1^H *T_1ρ_*** |
| **Figure** | **S1A** | **S1C** | **S1B** | **S2B** |
| ν_r_ / kHz | 17.0 | 17.0 | 100.0 | 17.0 |
| *B*_0_/ T | 16.4 | 16.4 | 16.4 | 16.4 |
| transfer I | ^1^H-^13^C CP | ^1^H-^15^N CP | ^1^H-^15^N | ^1^H spin lock |
| *ν*_1_(^1^H) / kHz | 60.0 | 60.0 | 70.0 | 45.0 |
| *ν*_1_(X) / kHz | 45.0 | 47.0 | 19.9 | - |
| Shape | Tangent ^1^H | Tangent ^1^H | Tangent ^1^H | - |
| time / ms | 0.5 | 0.75 | 1.0 | - |
| transfer II | DARR | ^15^N-^13^C CP | ^15^N-^1^H CP | ^1^H-^13^C CP |
| *ν*_1_(^1^H) / kHz | 17.0 | - | 70.0 | 60.0 |
| *ν*_1_(^15^N) / kHz | - | 9.9 | 19.9 | - |
| *ν*_1_(^13^C) / kHz | - | 12.0 | - | 44.7 |
| Shape | - | Tangent ^13^C | Tangent ^1^H | Tangent ^1^H |
| time / ms | 20.0 | 5.0 | 1.0 | 0.5 |
| *t_1_* increments | 2560 | 1536 | 512 | - |
| spectral width (*t_1_*) / kHz | 100.0 | 66.7 | 100.0 | - |
| acquisition time (*t_1_*) / ms | 12.8 | 11.5 | 25.6 | - |
| *t_2_* increments | 3072 | 3072 | 16384 | 3072 |
| spectral width (*t_2_*) / kHz | 100.0 | 100.0 | 100.0 | 100.0 |
| acquisition time (*t_2_*) / ms | 15.4 | 15.4 | 81.9 | 15.4 |
| Decoupling sequence | Spinal64 | Spinal64 | Swft-TPPM | Spinal64 |
| ^1^H decoupling power / kHz | 90.0 | 90.0 | 10.0 | 90.0 |
| interscan delay / s | 2.7 | 3.0 | 2.7 | 2.7 |
| number of scans | 32 | 8 | 32 | 64 |

**Table S6 continued:**

| **Sample** | **SmsC UL ^13^C/^15^N** | **DnaB UL ^13^C/^15^N : DNA : ADPAlF_4_***^–^* | **DnaB UL ^13^C/^15^N : DNA : ADPAlF_4_***^–^* | **SmsC : AMPPNP 1:50 day 4** |
| --- | --- | --- | --- | --- |
| **Experiment** | **^13^C *T_1ρ_*** | **^1^H *T_1ρ_*** | **^13^C *T_1ρ_*** | **^1^H-^31^P CP** |
| **Figure** | **S2A** | **S2B** | **S2A** | **1A, S3A, S4A** |
| ν_r_ / kHz | 17.0 | 17.0 | 17.0 | 17.0 |
| *B*_0_/ T | 16.4 | 16.4 | 16.4 | 11.7 |
| transfer I | ^1^H-^13^C CP | ^1^H spin lock | ^1^H-^13^C spin lock | ^1^H-^31^P CP |
| *ν*_1_(^1^H) / kHz | 60.0 | 45.0 | 60.0 | 60.0 |
| *ν*_1_(X) / kHz | 43.1 | - | 43.9 | 82.3 |
| Shape | Tangent ^1^H | - | Tangent ^1^H | Tangent ^1^H |
| time / ms | 0.4 | - | 0.6 | 1.5 |
| transfer II | ^13^C spin lock | ^1^H-^13^C CP | ^13^C spin lock | - |
| *ν*_1_(^1^H) / kHz | - | 60.0 | - | - |
| *ν*_1_(^13^C) / kHz | 45.0 | 43.0 | 45.0 | - |
| Shape | - | Tangent ^1^H | - | - |
| time / ms | - | 0.6 | - | - |
| *t_2_* increments | 3072 | 3072 | 3072 | 3072 |
| spectral width (*t_2_*) / kHz | 100.0 | 100.0 | 100.0 | 100.0 |
| acquisition time (*t_2_*) / ms | 15.4 | 15.4 | 15.4 | 15.4 |
| Decoupling sequence | Spinal64 | Spinal64 | Spinal64 | Spinal64 |
| ^1^H decoupling power / kHz | 90.0 | 90.0 | 90.0 | 90.0 |
| interscan delay / s | 2.7 | 2.7 | 2.7 | 2.7 |
| number of scans | 64 | 64 | 64 | 4096 |

**Table S6 continued:**

| **Sample** | **SmsC : AMPPNP 1:50 day 52** | **SmsC AMPPCP 1:50 day 2** | **SmsC AMPPCP 1:50 day 75** | **SmsC AMPPNP 1:50 day 4** |
| --- | --- | --- | --- | --- |
| **Experiment** | **^1^H-^31^P CP** | **^1^H-^31^P CP** | **^1^H-^31^P CP** | **^31^P-^31^P DARR** |
| **Figure** | **S4A** | **1A, S4B** | **S4B** | **1B** |
| ν_r_ / kHz | 17.0 | 17.0 | 17.0 | 17.0 |
| *B*_0_/ T | 11.7 | 11.7 | 11.7 | 11.7 |
| transfer I | ^1^H-^31^P CP | ^1^H-^31^P CP | ^1^H-^31^P CP | ^1^H-^31^P CP |
| *ν*_1_(^1^H) / kHz | 60.0 | 60.0 | 60.0 | 60.0 |
| *ν*_1_(X) / kHz | 69.2 | 55.9 | 80.2 | 82.3 |
| Shape | Tangent ^1^H | Tangent ^1^H | Tangent ^1^H | Tangent ^1^H |
| time / ms | 1.5 | 2.0 | 2.25 | 1.5 |
| transfer II | - | - | - | DARR |
| *ν*_1_(^1^H) / kHz | - | - | - | 17.0 |
| *t_1_* increments | - | - | - | 2048 |
| spectral width (*t_1_*) / kHz | - | - | - | 100.0 |
| acquisition time (*t_1_*) / ms | - | - | - | 10.2 |
| *t_2_* increments | 3072 | 1024 | 3072 | 2048 |
| spectral width (*t_2_*) / kHz | 100.0 | 100.0 | 100.0 | 100.0 |
| acquisition time (*t_2_*) / ms | 15.4 | 5.1 | 15.4 | 10.2 |
| Decoupling sequence | Spinal64 | Spinal64 | Spinal64 | Spinal64 |
| ^1^H decoupling power / kHz | 90.0 | 90.0 | 90.0 | 90.0 |
| interscan delay / s | 2.7 | 2 | 2.7 | 2.7 |
| number of scans | 14336 | 28672 | 24576 | 16 |

**Table S6 continued:**

| **Sample** | **SmsC : ADPAlF_4_***^–^* **1:50 day 4** | **SmsC : ADPAlF_4_***^–^* **1:50 day 4** | **SmsC : ADP 1:18 day 4** | **SmsC : ADP 1:18 day 32** |
| --- | --- | --- | --- | --- |
| **Experiment** | **^1^H-^31^P CP** | **^31^P-^31^P DARR** | **^1^H-^31^P CP** | **^1^H-^31^P CP** |
| **Figure** | **1A** | **S5** | **1A, S7, S8A** | **S8A** |
| ν_r_ / kHz | 17.0 | 17.0 | 17.0 | 17.0 |
| *B*_0_/ T | 11.7 | 11.7 | 11.7 | 11.7 |
| transfer I | ^1^H-^31^P CP | ^1^H-^31^P CP | ^1^H-^31^P CP | ^1^H-^31^P CP |
| *ν*_1_(^1^H) / kHz | 60.0 | 60.0 | 60.0 | 60.0 |
| *ν*_1_(X) / kHz | 77.8 | 77.8 | 77.2 | 77.2 |
| Shape | Tangent ^1^H | Tangent ^1^H | Tangent ^1^H | Tangent ^1^H |
| time / ms | 2.25 | 2.25 | 2.5 | 2.5 |
| transfer II | - | DARR | - | - |
| *ν*_1_(^1^H) / kHz | - | 17.0 | - | - |
| *t_1_* increments | - | 1024 | - | - |
| spectral width (*t_1_*) / kHz | - | 50.0 | - | - |
| acquisition time (*t_1_*) / ms | - | 10.2 | - | - |
| *t_2_* increments | 3072 | 3072 | 1024 | 3072 |
| spectral width (*t_2_*) / kHz | 100.0 | 100.0 | 100.0 | 100.0 |
| acquisition time (*t_2_*) / ms | 15.4 | 15.4 | 5.1 | 15.4 |
| Decoupling sequence | Spinal64 | Spinal64 | Spinal64 | Spinal64 |
| ^1^H decoupling power / kHz | 90.0 | 90.0 | 90.0 | 90.0 |
| interscan delay / s | 2.0 | 2.7 | 2.7 | 2.7 |
| number of scans | 3072 | 24 | 30720 | 30720 |

**Table S6 continued:**

| **Sample** | **SmsC : AMPCP 1:50 day 4** | **SmsC : AMPCP 1:50 day 23** | **SmsC : AMPCP 1:50 day 23** | **SmsC : AMPPNP 1:50 kinetic** |
| --- | --- | --- | --- | --- |
| **Experiment** | **^1^H-^31^P CP** | **^1^H-^31^P CP** | **^31^P DARR** | **^1^H-^31^P CP** |
| **Figure** | **1A, S8B** | **S8B** | **1C** | **4A** |
| ν_r_ / kHz | 17.0 | 17.0 | 17.0 | 17.0 |
| *B*_0_/ T | 11.7 | 11.7 | 11.7 | 11.7 |
| transfer I | ^1^H-^31^P CP | ^1^H-^31^P CP | ^1^H-^31^P CP | ^1^H-^31^P CP |
| *ν*_1_(^1^H) / kHz | 60.0 | 60.0 | 60.0 | 60.0 |
| *ν*_1_(X) / kHz | 72.2 | 72.2 | 72.2 | 72.2 |
| Shape | Tangent ^1^H | Tangent ^1^H | Tangent ^1^H | Tangent ^1^H |
| time / ms | 1.5 | 1.5 | 1.5 | 1.5 |
| transfer II | - | - | DARR | - |
| *ν*_1_(^1^H) / kHz | - | - | 17.0 | - |
| time / ms | - | - | 200.0 | - |
| *t_1_* increments |  |  | 2048 |  |
| spectral width (*t_1_*) / kHz | - | - | 100.0 | - |
| acquisition time (*t_1_*) / ms | - | - | 10.2 | - |
| *t_2_* increments | 3072 | 3072 | 2048 | 3072 |
| spectral width (*t_2_*) / kHz | 100.0 | 100.0 | 100.0 | 100.0 |
| acquisition time (*t_2_*) / ms | 15.4 | 15.4 | 10.2 | 15.4 |
| Decoupling sequence | Spinal64 | Spinal64 | Spinal64 | Spinal64 |
| ^1^H decoupling power / kHz | 90.0 | 90.0 | 90.0 | 90.0 |
| interscan delay / s | 2.7 | 2.7 | 2.7 | 2.7 |
| number of scans | 21504 | 9216 | 32 | 1024 |

**Table S6 continued:**

| **Sample** | **SmsC : AMPPNP kinetic** | **SmsC : AMPPNP 1:50** | **SmsC : ADPAlF_4_***^–^* **1:50** | **SmsC : AMPCP 1:50** |
| --- | --- | --- | --- | --- |
| **Experiment** | **^31^P CT-DRENAR** | **^31^P CT-DRENAR** | **^31^P CT-DRENAR** | **^31^P CT-DRENAR** |
| **Figure** | **4B** | **S12** | **3A** | **3B** |
| ν_r_ / kHz | 17.0 | 17.0 | 17.0 | 17.0 |
| *B*_0_/ T | 11.7 | 11.7 | 11.7 | 11.7 |
| transfer I | ^1^H-^31^P CP | ^1^H-^31^P CP | ^1^H-^31^P CP | ^1^H-^31^P CP |
| *ν*_1_(^1^H) / kHz | 40.0 | 60.0 | 60.0 | 60.0 |
| *ν*_1_(X) / kHz | 58.0 | 82.3 | 78.2 | 78.9 |
| Shape | Tangent ^1^H | Tangent ^1^H | Tangent ^1^H | Tangent ^1^H |
| time / ms | 2.25 | 1.5 | 2.25 | 2.25 |
| transfer II | Baba DQ excitation | Baba DQ excitation | Baba DQ excitation | Baba DQ excitation |
| *ν*_1_(^31^P) / kHz | 50.0 | 50.0 | 50.0 | 50.0 |
| time / ms | 1.9 | 1.9 | 1.9 | 1.9 |
| *t_2_* increments | 3072 | 3072 | 3072 | 3072 |
| spectral width (*t_2_*) / kHz | 100.0 | 100.0 | 100.0 | 100.0 |
| acquisition time (*t_2_*) / ms | 15.4 | 15.4 | 15.4 | 15.4 |
| Decoupling sequence | Spinal64 | Spinal64 | Spinal64 | Spinal64 |
| ^1^H decoupling power / kHz | 90.0 | 90.0 | 90.0 | 90.0 |
| interscan delay / s | 2.7 | 2.7 | 2.6 | 2.7 |
| number of scans | 2048 | 2048 | 1024 | 2048 |

**Table S6 continued:**

| **Sample** | **SmsC : AMPCP 1:50** | **SmsC : ADPAlF_4_***^–^* **1:50** | **SmsCB : AMPPNP 1:18** | **SmsCB : ADP 1:18** |
| --- | --- | --- | --- | --- |
| **Experiment** | **^1^H-^31^P CP** | **^1^H-^31^P CP** | **^1^H-^31^P CP** | **^1^H-^31^P CP** |
| **Figure** | **S9** | **S10** | **S3** | **S7** |
| ν_r_ / kHz | 3.0 | 3.0 | 17.0 | 17.0 |
| *B*_0_/ T | 11.7 | 11.7 | 11.7 | 11.7 |
| transfer I | ^1^H-^31^P CP | ^1^H-^31^P CP | ^1^H-^31^P CP | ^1^H-^31^P CP |
| *ν*_1_(^1^H) / kHz | 52.6 | 50.0 | 59.3 | 59.3 |
| *ν*_1_(X) / kHz | 64.5 | 47.0 | 77.2 | 77.2 |
| Shape | Tangent ^1^H | Tangent ^1^H | Tangent ^1^H | Tangent ^1^H |
| time / ms | 2.25 | 2.25 | 2.5 | 2.5 |
| *t_2_* increments | 3072 | 3072 | 1024 | 3072 |
| spectral width (*t_2_*) / kHz | 100.0 | 100.0 | 100.0 | 100.0 |
| acquisition time (*t_2_*) / ms | 15.4 | 15.4 | 5.1 | 15.4 |
| Decoupling sequence | Spinal64 | Spinal64 | Spinal64 | Spinal64 |
| ^1^H decoupling power / kHz | 90.0 | 90.0 | 90.0 | 90.0 |
| interscan delay / s | 2.7 | 2.7 | 2.7 | 2.7 |
| number of scans | 26624 | 21504 | 26624 | 18432 |

**Table S6 continued:**

| **Sample** | **SmsC : ADPAlF_4_***^–^* **1:50** | **DnaB UL ^13^C/^15^N : DNA : ADPAlF_4_***^–^* | **SmsCB:AMPPNP days 32 1:18** |
| --- | --- | --- | --- |
| **Experiment** | **^19^F EASY** | **^1^H-^31^P CP** | **^1^H-^31^P CP** |
| **Figure** | **S6** | **S11** | **S3** |
| ν_r_ / kHz | 17.0 | 3.0 | 17.0 |
| *B*_0_/ T | 11.7 | 11.7 | 11.7 |
| transfer I | ^19^F π/2 pulse | ^1^H-^31^P CP | ^1^H-^31^P CP |
| *ν*_1_(^1^H) / kHz | - | 50.8 | 59.3 |
| *ν*_1_(X) / kHz | 100.0 | 37.1 | 77.2 |
| Shape | - | Tangent ^1^H | Tangent ^1^H |
| time / ms | - | 2.25 | 2.5 |
| *t_2_* increments | 16384 | 3072 | 3072 |
| spectral width (*t_2_*) / kHz | 714.3 | 100.0 | 100.0 |
| acquisition time (*t_2_*) / ms | 11.5 | 15.4 | 15.4 |
| Decoupling sequence | - | Spinal64 | Spinal64 |
| ^1^H decoupling power / kHz | - | 90.0 | 90.0 |
| interscan delay / s | 2.5 | 2.5 | 2.7 |
| number of scans | 2048 | 21504 | 26624 |

**Supplementary references**

[1]	O. H. Lowry, N. J. Rosebrough, A. L. Farr, R. J. Randall, “Protein measurement with the Folin Phenol reagent” *J. Biol. Chem.* **1951**, *193*, 265–75.

[2]	J. H. Miller, *Experiments in molecular genetics*, Cold Spring Harbor Laboratory, Cold Spring Harbor, NY, **1972**.

[3]	C. Gardiennet, A. K. Schütz, A. Hunkeler, B. Kunert, L. Terradot, A. Böckmann, B. H. Meier, “A sedimented sample of a 59 kDa dodecameric helicase yields high-resolution solid-state NMR spectra” *Angew. Chem. Int. Ed.* **2012**, *51*, 7855–7858.

[4]	T. Wiegand, R. Cadalbert, D. Lacabanne, J. Timmins, L. Terradot, A. Böckmann, B. H. Meier, “The conformational changes coupling ATP hydrolysis and translocation in a bacterial DnaB helicase” *Nat. Commun.* **2019**, *10*, 31.

[5]	C. R. Corbeil, C. I. Williams, P. Labute, “Variability in docking success rates due to dataset preparation” *J. Comput. Aided Mol. Des.* **2012**, *26*, 775–786.

[6]	T. Le Marchand, T. Schubeis, M. Bonaccorsi, P. Paluch, D. Lalli, A. J. Pell, L. B. Andreas, K. Jaudzems, J. Stanek, G. Pintacuda, “1H-Detected biomolecular NMR under fast magic-angle spinning” *Chem. Rev.* **2022**, *122*, 9943–10018.

[7]	U. Sternberg, R. Witter, I. Kuprov, J. M. Lamley, A. Oss, J. R. Lewandowski, A. Samoson, “1H line width dependence on MAS speed in solid state NMR – Comparison of experiment and simulation” *J. Magn. Reson.* **2018**, *291*, 32–39.

[8]	S. Penzel, A. Oss, M. L. Org, A. Samoson, A. Böckmann, M. Ernst, B. H. Meier, “Spinning faster: protein NMR at MAS frequencies up to 126 kHz” *J. Biomol. NMR* **2019**, *73*, 19–29.

[9]	T. Wiegand, M. Schledorn, A. A. Malär, R. Cadalbert, A. Däpp, L. Terradot, B. H. Meier, A. Böckmann, “Nucleotide binding modes in a motor protein revealed by 31P- and 1H-detected MAS solid-state NMR spectroscopy” *ChemBioChem* **2020**, *21*, 324–330.

[10]	Z. Sun, C. Ollier, A. Rancz, B. Thienpont, K. Grohe, L. Becker, A. Purea, F. Engelke, S. Wegner, J. Sturgis, T. Polenova, T. Le Marchand, G. Pintacuda, “Pushing the boundaries of resolution in solid-state nuclear magnetic resonance of biomolecules with 160 kHz magic-angle spinning” *J. Am. Chem. Soc.* **2025**, *147*, 19433–19437.

[11]	D. Cala-De Paepe, J. Stanek, K. Jaudzems, K. Tars, L. B. Andreas, G. Pintacuda, “Is protein deuteration beneficial for proton detected solid-state NMR at and above 100 kHz magic-angle spinning?” *Solid State Nucl. Magn. Reson.* **2017**, *87*, 126–136.

[12]	A. Torosyan, T. Wiegand, M. Schledorn, D. Klose, P. Güntert, A. Böckmann, B. H. Meier, “Including protons in solid-state NMR resonance assignment and secondary structure analysis: The example of RNA Polymerase II subunits Rpo4/7” *Front. Mol. Biosci.* **2019**, *6*, DOI 10.3389/fmolb.2019.00100.

[13]	J. Struppe, C. M. Quinn, M. Lu, M. Wang, G. Hou, X. Lu, J. Kraus, L. B. Andreas, J. Stanek, D. Lalli, A. Lesage, G. Pintacuda, W. Maas, A. M. Gronenborn, T. Polenova, “Expanding the horizons for structural analysis of fully protonated protein assemblies by NMR spectroscopy at MAS frequencies above 100 kHz” *Solid State Nucl. Magn. Reson.* **2017**, *87*, 117–125.

[14]	D. Lacabanne, J. Boudet, A. A. Malär, P. Wu, R. Cadalbert, L. Salmon, F. H. T. Allain, B. H. Meier, T. Wiegand, “Protein side-chain-DNA contacts probed by fast magic-angle spinning NMR” *J. Phys. Chem. B* **2020**, *124*, 11089–11097.

[15]	I. Bertini, C. Luchinat, G. Parigi, E. Ravera, B. Reif, P. Turano, “Solid-state NMR of proteins sedimented by ultracentrifugation” *Proc. Natl. Acad. Sci. U. S. A.* **2011**, *108*, 10396–10399.

[16]	Y. Zi Tan, P. R. Baldwin, J. H. Davis, J. R. Williamson, C. S. Potter, B. Carragher, D. Lyumkis, “Addressing preferred specimen orientation in single-particle cryo-EM through tilting” *Nat. Methods* **2017**, *14*, 793–796.

[17]	D. Lyumkis, “Challenges and opportunities in cryo-EM single-particle analysis” *J. Biol. Chem.* **2019**, *294*, 5181–5197.

[18]	R. M. Glaeser, “How good can cryo-EM become” *Nat. Methods* **2016**, *13*, 28–32.

[19]	M. J. Bayro, M. Huber, R. Ramachandran, T. C. Davenport, B. H. Meier, M. Ernst, R. G. Griffin, “Dipolar truncation in magic-angle spinning NMR recoupling experiments” *J. Chem. Phys.* **2009**, *130*, 114506.

[20]	J. Ren, H. Eckert, “DQ-DRENAR: A new NMR technique to measure site-resolved magnetic dipole-dipole interactions in multispin-12 systems: Theory and validation on crystalline phosphates” *J. Chem. Phys.* **2013**, *138*, 164201.

[21]	J. Ren, H. Eckert, “Applications of DQ-DRENAR for the structural analysis of phosphate glasses” *Solid State Nucl. Magn. Reson.* **2015**, *72*, 140–147.

[22]	J. Ren, H. Eckert, “DQ-DRENAR with back-to-back (BABA) excitation: Measuring homonuclear dipole-dipole interactions in multiple spin-1/2 systems” *Solid State Nucl. Magn. Reson.* **2015**, *71*, 11–18.

[23]	J. Ren, H. Eckert, “Measurement of homonuclear magnetic dipole-dipole interactions in multiple 1/2-spin systems using constant-time DQ-DRENAR NMR” *J. Magn. Reson.* **2015**, *260*, 46–53.

[24]	U. Haeberlen, *High resolution NMR in solids: selective averaging. Advances in magnetic resonance supplement 1*, Academic Press, **1976**.

[25]	M. Mehring, *High Resolution NMR Spectroscopy in Solids*, Springer Berlin, Heidelberg, **1976**.

[26]	A. Steigel, H. W. Spiess, *Dynamic NMR Spectroscopy*, Springer Verlag, Berlin, Heidelberg, New York, **1978**.

[27]	S. Hediger, B. H. Meier, D. K. Narayanan, G. Bodenhausen, R. R. Ernst, “NMR cross polarization by adiabatic passage through the Hartmann-Hahn condition (APHH)” *Chem. Phys. Lett.* **1994**, *223*, 283–288.
